# Hepatic segmental arterial mediolysis: A case report and brief literature review

**DOI:** 10.1002/ccr3.7668

**Published:** 2023-07-10

**Authors:** Ashbina Pokharel, Ioannis Karageorgiou, Sangam Shah, Madhur Bhattarai, Indira Acharya, Judith Bateman

**Affiliations:** ^1^ Department of Internal Medicine William Beaumont University Hospital Royal Oak Michigan USA; ^2^ Tribhuwan University, Institute of Medicine Kathmandu Nepal; ^3^ Department of Internal Medicine, Medstar Union Memorial Hospital Baltimore Maryland USA; ^4^ Department of Rheumatology William Beaumont University Hospital Royal Oak Michigan USA

**Keywords:** hepatic, SAM, segmental arterial mediolysis

## Abstract

**Key Clinical message:**

When evaluating patients with abdominal pain, it is important to consider SAM in the differential diagnosis, along with vasculitis, fibromuscular dysplasia (FMD), atherosclerosis, mycotic aneurysms, and cystic medial degeneration.

**Abstract:**

Segmental arterial mediolysis (SAM) is a rare arteriopathy which is an under‐recognized and commonly missed diagnosis of abdominal pain. We report a case of a 58‐year‐old female who presented with abdominal pain and was misdiagnosed with a urinary tract infection. The diagnosis was made with CTA and managed with embolization. Despite appropriate intervention and close hospital monitoring, further complications were inevitable. We conclude that though literature has shown better prognosis and even complete resolution after medical and/or surgical intervention, close follow up and monitoring is needed to avoid unexpected complications.

## 1 INTRODUCTION

Segmental arterial Mediolysis (SAM) is a non‐atherosclerotic, non‐inflammatory vasculopathy of unknown etiology characterized by vacuolization and lysis of outer media layer of artery leading to stricture, aneurysm, occlusion, and dissection.^1^, ^2^, ^3^ It usually involves medium and large sized abdominal splanchnic vessels, such as the celiac, mesenteric, and/or renal arteries with occasional carotid, cerebral, and coronary artery involvement.^2^ The usual presentation is a middle‐aged or elderly patient with abdominal and/or flank pain but could also present with catastrophic hypovolemia or hemorrhagic shock in severe forms.^4^ Although histology is still considered the standard method for confirming a diagnosis of SAM, the growing quality of non‐invasive imaging techniques such as CT and MR angiograms has led to an increase in the use of these imaging modalities over tissue biopsy.^2^, ^3^ This shift is due to current discrepancies regarding anatomic involvement and the use of inflammatory markers, autoimmune serologies, and genetic testing necessary to diagnose SAM.^2^, ^3^, ^5^ Moreover, there is significant overlap between SAM and similar arteriopathies such as an aneurysms, dissections, and fibromuscular dysplasia.^2^, ^5^ Therefore, it is necessary to standardize the diagnostic criteria for SAM and its mimics. Though there have been reports of complete resolution of vascular lesion of SAM or long‐term disease‐free survival following embolization, bypass or resection of affected areas, cases complicated by abdominal hemorrhage had a mortality of 50%,^6^ which has been reduced to 25% with the introduction of endovascular interventions.^3^ Given its high mortality and emergent presentation, a high degree of suspicion for diagnosis is required to avoid delays in treatment. Here we present a case of 58‐year‐old woman with a delayed diagnosis of SAM that led to rapid deterioration of her clinical status despite aggressive measures. This case highlights how SAM could be repeatedly misdiagnosed and how a patient could deteriorate quickly emphasizing the importance of clinical judgment.

## 2 CASE PRESENTATION

A 58‐year‐old female with a past medical history of protein C and S deficiency, deep vein thrombosis (on Warfarin), systemic lupus erythematosus and hypothyroidism presented to an urgent care with right flank and upper quadrant abdominal pain for 3 days. Urinalysis revealed blood and leukocyte esterase and the patient was discharged on Cephalexin for suspected urinary tract infection. Her abdominal pain did not improve, and she started having nausea, prompting her to visit the emergency department. In the ED, a Computed Tomography (CT) of her abdomen showed findings consistent with recently passed calculus and the patient was again discharged home with a plan for outpatient follow up.

She returned after 12 h as her abdominal pain got worse. Her vital signs at presentation were blood pressure 154/80 mm Hg, heart rate 85 beats per min, respiratory rate 17 per min and oxygen saturation of 100% on room air. Physical examination revealed diffuse abdominal tenderness along with right costovertebral angle tenderness. Her initial laboratory test results showed white blood cell count 9.1 bil/L, hemoglobin 13 g/dL, platelets 257 bil/L and INR 4.4. Basic metabolic panel was unremarkable. Liver function panel showed aspartate aminotransferase 658 U/L (compared to 104 U/L 12 h prior), alanine aminotransferase 772 U/L (compared to 82 U/L 12 h prior), bilirubin 0.5 mg/dL, alkaline phosphatase 70 U/L and albumin of 4.6 g/dL. Extensive workup including infectious and rheumatologic testing to understand the etiology of acute elevation in transaminases was grossly unremarkable as in Table 1.

**TABLE 1:** Laboratory results.

Variable	Result	Reference range
Erythrocyte sedimentation rate	73	0–18 mm/hr
Anti neutrophil cytoplasmic antibody	<1:20	<1:20
Total complement	>95	> = 41.7 U/mL
Complement C4	19 mg/dL	10–43 mg/dL
Myeloperoxidase antibody	3 U	<=20 U
Proteinase 3 antibody	3 U	<=20 U
Anti cardiolipin antibody, IgA	<9.5	<12 APL
Anti cardiolipin antibody, IgG	<9.5	<15 GPL
Anti cardiolipin antibody, IgM	9.6	<12.5 MPL
Beta 2 glycoprotein1, IgG	<9.5	<=20 SGU
Beta 2 glycoprotein 1, IgM	<9.5	<=20 SMU
Hepatitis B surface antigen	Nonreactive	Nonreactive
Hepatitis B core antibody Total	Nonreactive	Nonreactive
Hepatitis C virus antibody	Nonreactive	Nonreactive

**FIGURE 1:**
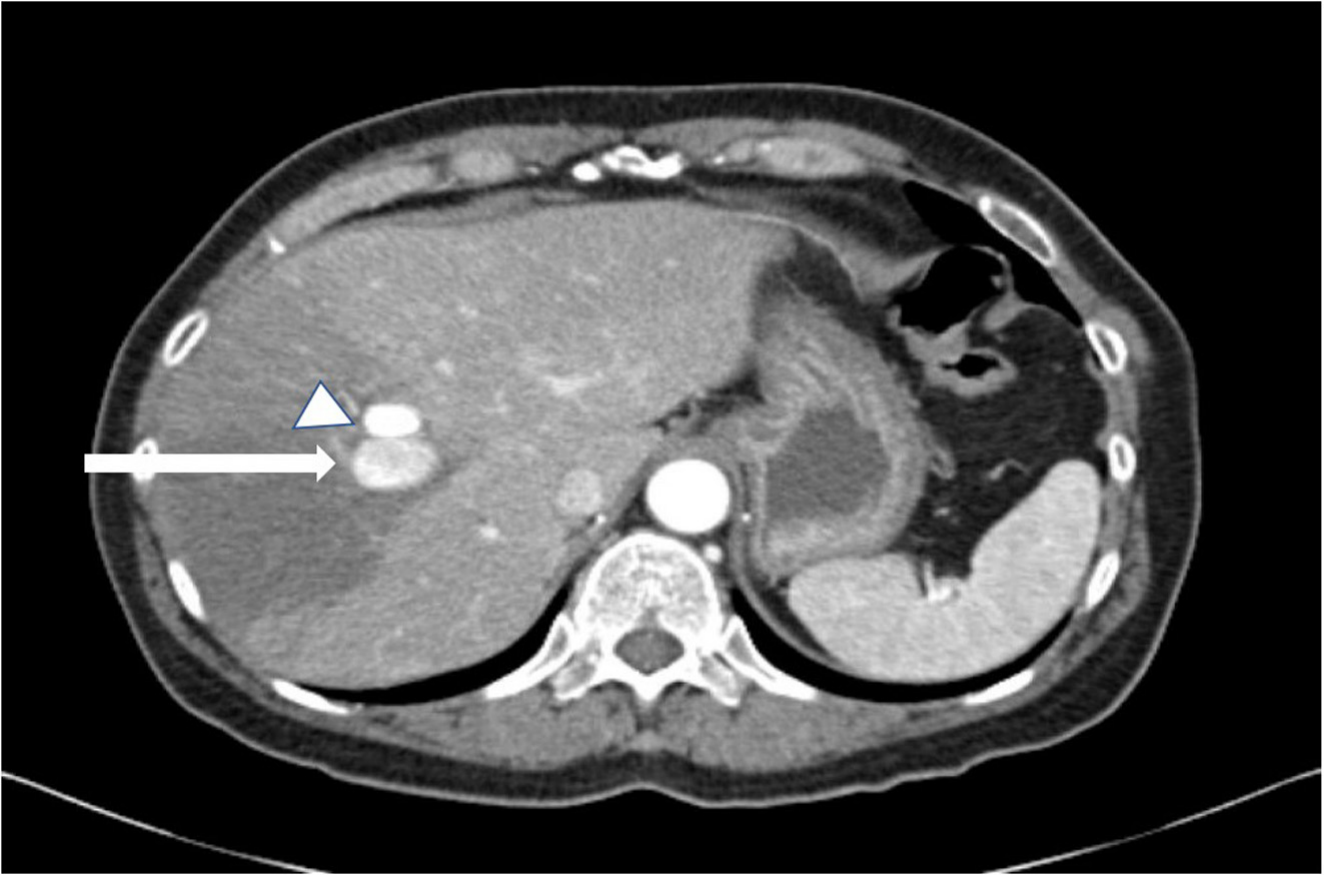
Axial contrast‐ enhanced computed tomography angiography showing pseudoaneurysms in right hepatic artery (arrow and arrow head).

**FIGURE 2:**
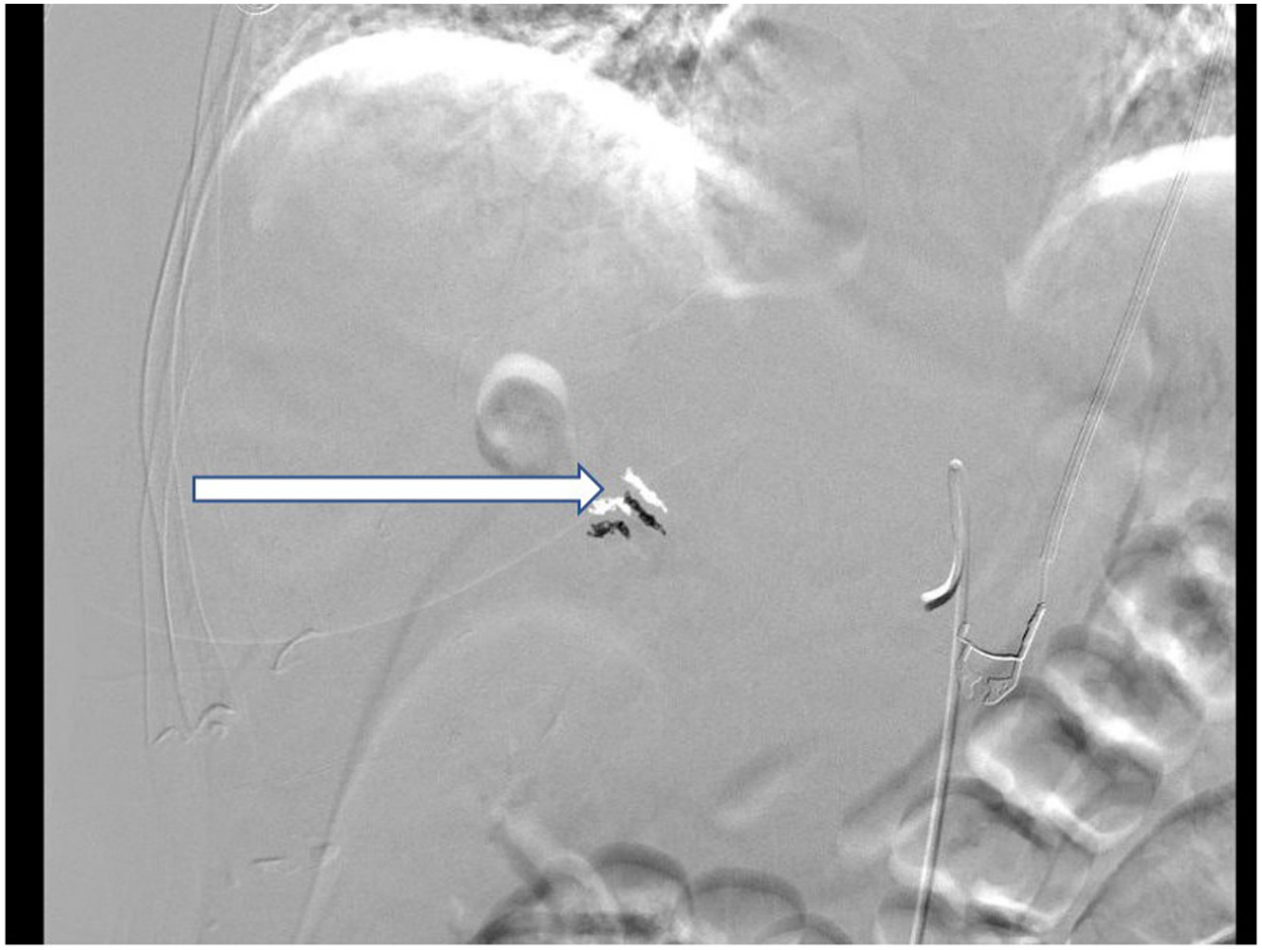
Angiogram image of right hepatic artery after coiling(arrow).

**FIGURE 3:**
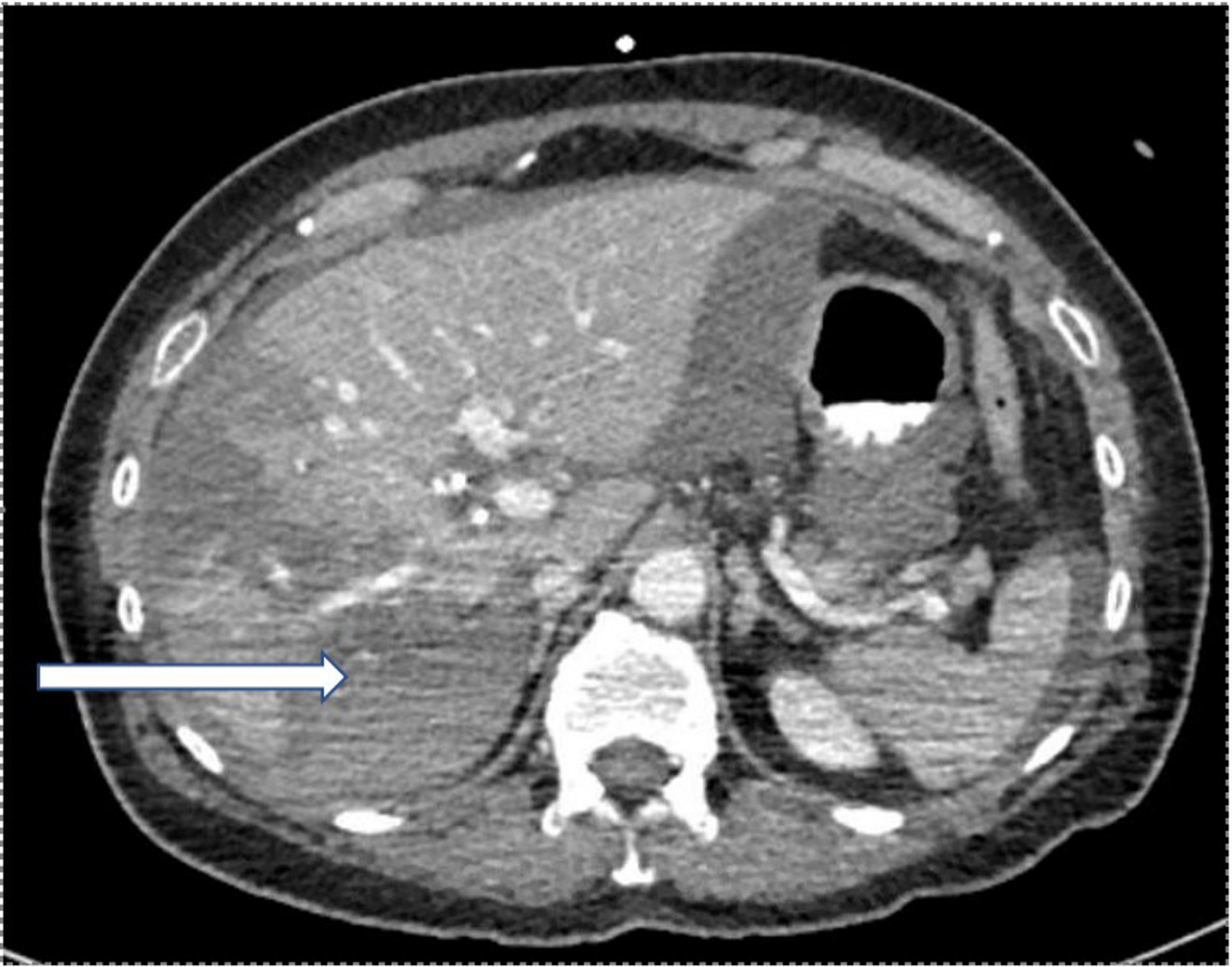
Axial contrast‐enhanced computed tomography showing perihepatic hematoma(arrow).

**FIGURE 4:**
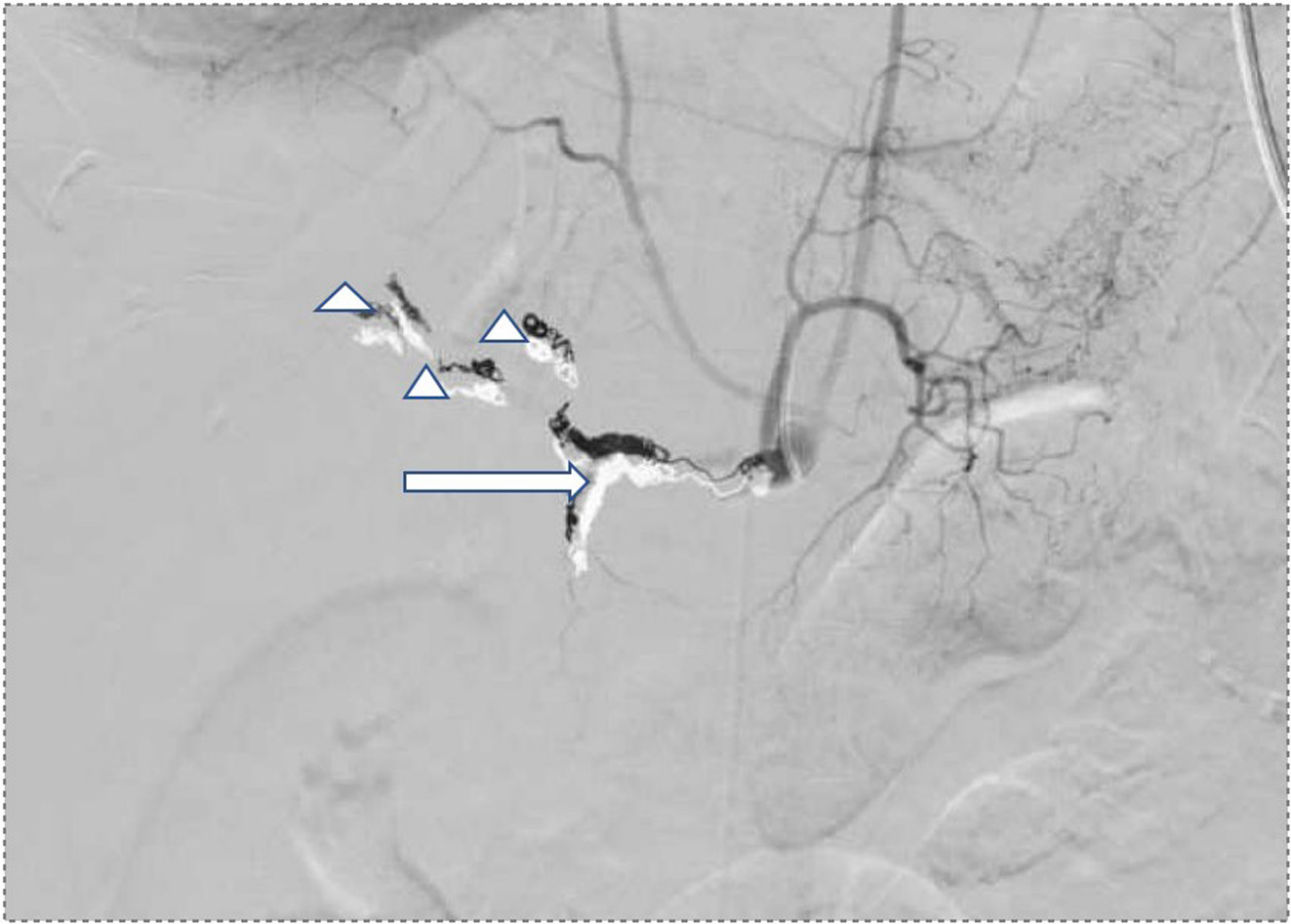
Angiogram images after coiling of common hepatic artery and gastroduodenal artery(arrow), right and left hepatic arteries(arrow head).

**FIGURE 5:**
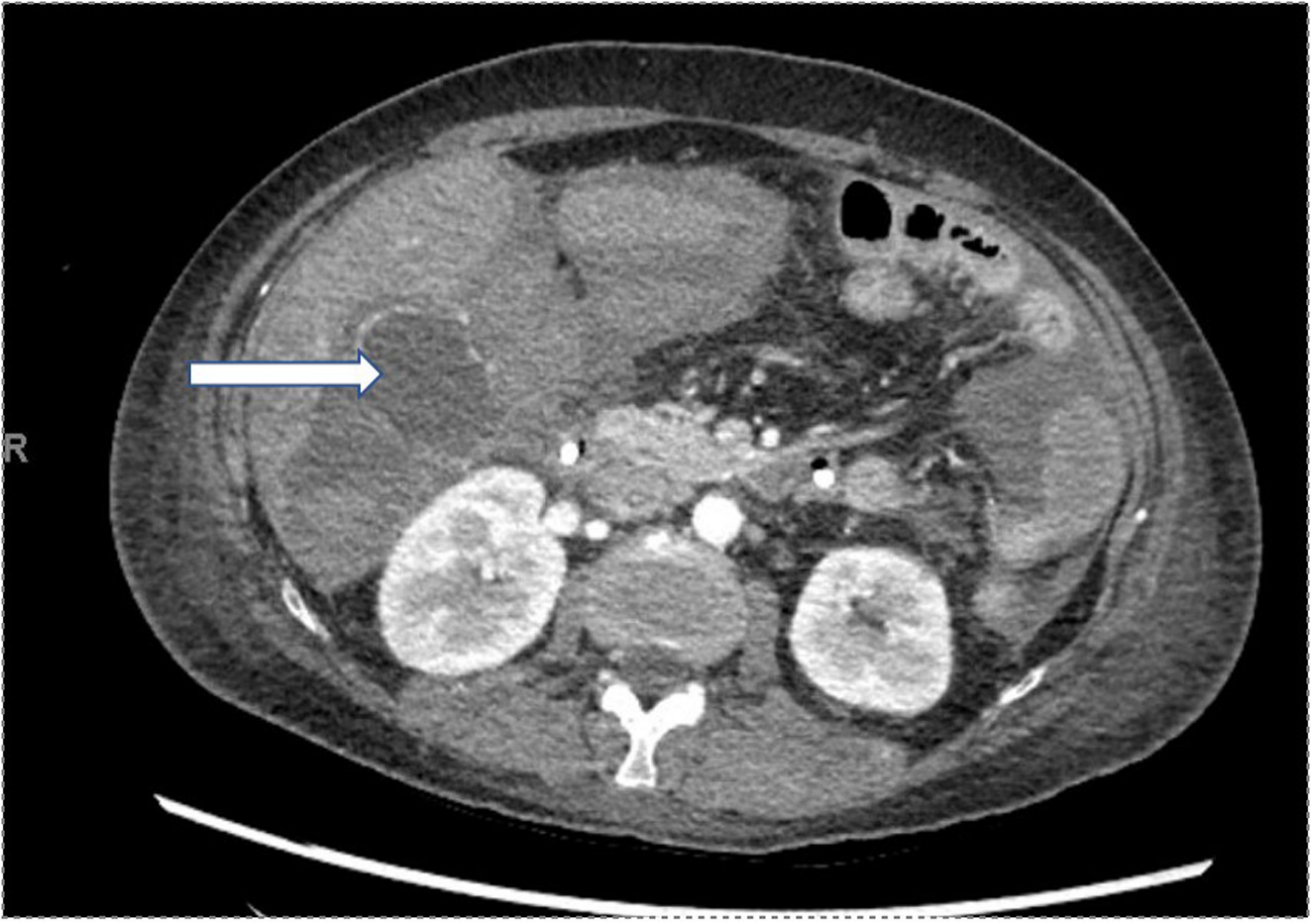
Axial contrast‐enhanced tomography showing perihepatic bilioma (arrow).

An axial CT angiography abdomen/ pelvis with contrast showed new right hepatic lobe infarct measuring 6.5 × 6.5 cm with hypervascular lesion suspicious for pseudoaneurysm of right hepatic artery as in Figure 1 and occlusion of the anterior right portal vein at the site of aneurysm resulting in anterior right hepatic lobe infarction (Figure 1). An MRI of her abdomen showed a wedge‐shaped hepatic ischemia to portions of segment 5/8 likely secondary to a 3 cm intrahepatic pseudoaneurysm with compression of anterior branch of right portal vein. The clinical history and angiographic findings were strongly suggestive of a diagnosis of SAM. The patient subsequently underwent embolization of hepatic pseudoaneurysm with gelfoam and coil embolization as in Figure 2 and started to show symptomatic improvement. On day 5 of hospitalization, the patient suddenly developed one episode of syncope and diffuse abdominal pain. Lab results were remarkable for a marked hemoglobin drop to 5.2 g/dL (compared to 9.6 g/dL a day prior) and accordingly, the patient underwent transfusion. CT angiogram abdomen/pelvis was repeated which showed large perihepatic hematoma (Figure 3) with enhancing structure in right hepatic lobe near site of prior embolization which was concerning for re‐bleeding of the pseudoaneurysm and extravasation along with new moderate volume of hemoperitoneum. Subsequently, the patient underwent repeat embolization of the pseudoaneurysm, common hepatic artery, right hepatic artery, medial branch of left hepatic artery and gastroduodenal artery as in Figure 4. She was monitored closely in the surgical intensive care unit and serial monitoring of hemoglobin and liver enzymes was done. Patient reported persistent abdominal pain and subsequently developed frequent loose stools and fever with a maximum temperature of 101.4 F. Clostridium difficile testing was negative and a repeat CT abdomen showed gallbladder wall rupture with large perihepatic biloma (Figure 5) extending to right paracolic gutter along with perihepatic hematoma extending to right paracolic gutter, infarction of right hepatic lobe and a small splenic infarction. The patient accordingly underwent exploratory laparotomy and a cholecystectomy along with drainage of the intra‐abdominal hematoma. She continued to have fever spikes despite being on intravenous broad‐spectrum antibiotics (Vancomycin and Piperacillin‐Tazobactam). Four days later the patient suddenly became hypoxic and went into cardiac arrest. Bedside emergent decompressive laparotomy was performed for suspected abdominal compartment syndrome. Bowel loops were found to be distended though perfusion was maintained, and no active bleeding or intra‐abdominal hematoma noted. Unfortunately, 3 days later the patient went into cardiac arrest again and subsequently passed away.

## 3 DISCUSSION

SAM was initially described by Slavin et al in 1976 as “Segmental mediolytic arteritis”.^1^ Since then, we know that it is a non‐inflammatory, non‐atherosclerotic vasculopathy that affects medium sized vessels, most commonly branches of the aorta.^7^ Multiple retrospective reviews, systematic reviews and meta‐analyses have been conducted to establish the epidemiology, prognosis, diagnostic criteria, as well as best treatment option for patients.^2^, ^3^, ^8^, ^9^, ^10^, ^11^ However, because the disease is mostly asymptomatic, as well as because it is rare, relatively few reports exist, with no large‐scale studies evaluating treatment outcomes.

The pathophysiology of SAM has been the topic of extensive study and is mainly guided by histologic findings.^5^, ^7^, ^12^ It is theorized that development of vacuoles in the smooth muscle of the outer media of arterial walls (mediolysis) is the first step, followed by granulation tissue formation and fibrosis that leads to the eventual disappearance of the vacuole if rupture does not occur between these two steps.^5^, ^7^ Slavin et al, more specifically has mentioned that SAM can be described as a vasospastic arteriopathy that is triggered when norepinephrine binds with alpha‐1 adrenoceptor on the vessel walls. As the arteriopathy is repaired, it is converted into various standardized arterial diseases that change the clinical presentation of SAM from a hemorrhagic disorder to an ischemic one.^12^ Moreover, one of the histologic hallmarks of SAM is absence of inflammatory cells.^5^, ^7^

Most studies concur that SAM most commonly affects men aged 50–60. ^3^, ^8^, ^9^, ^10^, ^11^ However, the disease has been reported in all age groups, including neonates.^3^, ^5^ No comorbidities have been associated with SAM^3^, but most studies, reported hypertension in a significant number of patients with the disease.^2^, ^3^ However, it is possible that this finding reflects the incidence of hypertension in this population.^3^ Other reported associations include hyperlipidemia and tobacco use.^2^ The natural course of unruptured aneurysms in over two thirds of patients with SAM is stabilization of the aneurysm during follow up.^10^ The rest of the aneurysms will either reduce in size or disappear during follow up^10^. A small percentage of patients (4% of patients with unruptured aneurysms) will develop new aneurysms according to Shimohira et al.^10^ Approximately 25% of patients present with aneurysm rupture,^10^ and will need immediate surgical intervention. Death in SAM usually occurs within 30 days of presentation.^3^

Most of the studies published agree on the most common presenting symptom being abdominal pain in over two thirds of cases.^7^, ^8^, ^9^, ^11^ Other less common presenting complaints include flank pain, and back pain, as well as no symptoms.^2^, ^9^, ^11^ In cases of aneurysmal rupture, hypotension has also been described at presentation.^9^ The most affected arteries are branches of the aorta, with different studies reporting different percentages. The splenic artery, celiac trunk, SMA, hepatic artery, and renal arteries are affected in most studies at different rates.^2^, ^3^, ^8^, ^9^, ^10^, ^11^ However, it is worth mentioning that more frequently than not, multiple different arteries are involved, or multiple sites in the same artery.^2^, ^8^, ^11^ In our case, the hepatic artery and its branches were involved, with no evidence of other arterial involvement.

Diagnosing SAM always poses a clinical challenge.^5^, ^7^ The gold standard for diagnosis is arterial biopsy, which shows the presence of vacuoles in the arterial media^5^, ^7^ with lack of inflammation. However, in most cases biopsy not practical or even impossible, due to the acuity of presentation or the location of the lesion. Diagnostic criteria (clinical, imaging, serologic) have therefore been developed that are used widely in the literature for the diagnosis of the disease,^2^, ^7^, ^9^ even though they are not universally agreed upon, and have been developed based on expert opinion. The clinical criterion is to rule out other causes that could cause similar presentation, including connective tissue diseases (Marfan syndrome), atherosclerosis, FMD, and other vasculitides.^7^, ^9^ In terms of imaging criteria, SAM usually presents with one of 6 presenting imaging findings, according to Slavin et al, which include dissection, fusiform aneurysm, saccular aneurysm, occlusion, stenosis, beaded appearance, wall thickening, pseudoaneurysm, fistula, or organ infarction. ^13^ CTA is being utilized increasingly for the diagnosis of SAM instead of angiography, due to its less invasive nature, and ability to assess the vessel wall as well.^6^, ^7^, ^11^ For the serologic criterion, inflammation also needs to be ruled out, with negative inflammatory markers. This is sometimes challenging, as elevated CRP can be seen in cases of acute illness, as reported in previous studies.^9^

Differential diagnosis of this disease is very important, as there are multiple mimics that could make diagnosing SAM particularly challenging.^2^, ^5^, ^7^, ^9^, ^13^ Atherosclerosis as a cause needs to be considered, but patients usually have diffuse atherosclerosis of multiple vessels, and the disease affects artery bifurcations.^5^ SAM is usually isolated in medium sized vessels and has no preference for bifurcations.^5^ Vasculitides need to be ruled out, which is usually done with the assistance of inflammatory markers, as well as the absence of clinical findings that support a systemic vasculitis.^5^ Marfan syndrome and cystic medial necrosis also are in the differential and can be ruled out by absence of characteristic clinical findings as well as different histology (cystic medial necrosis).^5^ Mycotic aneurysms also need to be considered, but the lack of systemic infection, and inflammation makes these types of aneurysms unlikely in cases of SAM^5^.

Fibromuscular dysplasia requires special mention, as it shares many similar features with SAM.^2^, ^5^, ^7^, ^13^ Many studies have examined the differences and similarities between these two conditions, concluding that the best way to differentiate between the two conditions are the patient demographics.^5^, ^13^ FMD commonly affects young females and usually presents with hypertension. SAM affects middle aged males and presents with abdominal pain or hemorrhage.^5^, ^7^, ^9^, ^11^, ^13^ FMD affects the renal arteries, the intracerebral arteries as well as the carotids. SAM affects arteries of the celiac trunk and intracerebral vessels with different incidence.^2^, ^5^, ^13^ It is particularly difficult to distinguish SAM from FMD in the renal arteries as mentioned in previous studies.^2^ In our case, the hepatic artery was involved, which is rarely the case in FMD.^5^ Histology is the most conclusive way to differentiate between the two conditions.^5^

In the background of medical history such as protein C and S deficiency, deep vein thrombosis, systemic lupus erythematosus, and hypothyroidism in our patient, it is possible that they contribute to vasculopathy. The prevalence of vasculitis in SLE is reported to be between 11% and 36%.^14^Although currently the patient's inflammatory disease is in remission as evident by laboratory report, the possibility of formation of pseudoaneurysm while the disease were active cannot be eliminated. This could have been ruled out if histopathology was performed, which is the limitation in our case. The clinical picture of sudden abdominal pain, involvement of arteries from the celiac axis, and imaging findings of multiple pseudoaneurysm are typical findings for SAM and would correlate with it. Multiple cases have been reported with similar findings in the literature as SAM. SLE can lead to aneurysm but multiple pseudoaneurysm still favors the diagnosis of SAM. The presented case can also be a secondary SAM as a rare complication of patient's underlying diseases.

Treatment of SAM is conservative with blood pressure control, anticoagulation, or antiplatelet therapy,^2^ and active surveillance with serial imaging in most cases that present with unruptured SAM^10^. Most patients will have stable disease, with some having complete resolution of SAM, whereas others will have aneurysm progression or development of new lesions.^10^ In cases presenting with rupture or in unruptured cases that require intervention due to reasons mentioned above, surgical intervention is required either with endovascular procedures or open surgery.^2^, ^3^, ^8^, ^9^, ^10^ Most recent published studies mention that endovascular techniques, with the most popular one being coil embolization, are preferred over surgery (79% of cases), with high success rate, minimal complications, and nearly zero mortality. ^2^, ^8^, ^9^, ^10^ Open surgical intervention is reported in 20% of patients^2^ with a higher mortality rate (9%).^3^ In most studies, like in our case, surgery was performed due to failure of endovascular procedures to control the bleeding.^3^, ^9^

## 4 CONCLUSION

SAM is a rare non‐inflammatory, non‐atherosclerotic disease of medium sized intra‐abdominal vessels, most commonly affecting men between ages of 50–60. The most common presenting symptom is abdominal pain, and it is usually diagnosed with CTA. Differential diagnosis should include vasculitis, FMD, atherosclerosis, mycotic aneurysms, and Cystic medial degeneration. Most patients presenting with unruptured lesions will need continued surveillance but will not require surgical intervention. Patients with disease progression as well as patients with rupture will need surgical intervention, either endovascular or open surgery, with mortality being around 25%. Clinicians need to have a high suspicion for the diagnosis of this disease, especially if presenting acutely.

## AUTHOR CONTRIBUTIONS

**Ashbina Pokharel:** Conceptualization; writing – original draft; writing – review and editing. **Ioannis Karageorgiou:** Writing – original draft; writing – review and editing. **SANGAM SHAH:** Writing – review and editing. **Madhur Bhattarai:** Writing – review and editing. **Indira Acharya:** Writing – review and editing. **Judith Bateman:** Writing – review and editing.

## CONFLICT OF INTEREST STATEMENT

Authors' have no conflict of interest to declare.

## CONSENT

Written informed consent was obtained from the patient to publish this report in accordance with the journal's patient consent policy.

## Data Availability

All the required information is available in the manuscript itself.
